# Microbial community dynamics in phyto-thermotherapy baths viewed through next generation sequencing and metabolomics approach

**DOI:** 10.1038/s41598-020-74586-9

**Published:** 2020-10-21

**Authors:** Elena Franciosi, Luca Narduzzi, Antonella Paradiso, Silvia Carlin, Kieran Tuohy, Alberto Beretta, Fulvio Mattivi

**Affiliations:** 1 Research and Innovation Centre, AgriFood Quality and Nutrition Department, Fondazione Edmund Mach (FEM), Via E. Mach 1, 38010 San Michele all’Adige, Italy; 2 Ospedale San Raffaele - Milano, Via Olgettina 60, 20132 Milan, Italy; 3 Department of Cellular, Computational and Integrative Biology – CIBIO, University of Trento, Via Sommarive, 9, 38123 Trento, Italy

**Keywords:** Biochemistry, Ecology, Microbiology, Molecular biology

## Abstract

Phyto-thermotherapy is a treatment consisting in immersing oneself in baths of self-heating alpine grass, to benefit of the heat and rich aromatic components released by the process. The aim of this study was to characterize the bacterial and fungal diversity of three phyto-thermal baths (PTB) performed in three different months, and to compare the data with the profile of the volatile organic compounds (VOCs) of the process. All the data collected showed that PTBs were structured in two stages: the first three days were characterised by an exponential rise of the temperature, a fast bacterial development, higher microbial diversity and higher concentrations of plant aliphatic hydrocarbons. The second stage was characterised by a stable high temperature, shrinkage of the microbial diversity with a predominance of few bacterial and fungi species and higher concentrations of volatiles of microbial origin. *Erwinia* was the dominant microbial species during the first stage and probably responsible of the self-heating process. In conclusion, PTBs has shown both similarities with common self-heating processes and important peculiarities such as the absence of pathogenic bacteria and the dominance of plant terpenoids with health characteristics among the VOCs confirming the evidence of beneficial effects in particular in the first three days.

## Introduction

Phyto-thermotherapy^1, 2^ also known as Phyto-balneotherapy^3^ consists of immersing people in phyto-thermal baths (PTB) containing freshly-cut mountain grass in hot fermentation for no more than 30 min. PTB is an ancient practice performed since the nineteenth century in mountain areas of Tyrol (Austria), Alto Adige and Trentino (Italy). The PTBs are usually located in Spa and loaded with mountain herbs cut at vegetative maturity. An historical area for PTB grass collection is the meadow of the Viote in Monte Bondone (1540 m a.s.l., Trentino, Italy). The grass is harvested during the summer season, cut and bailed in early mornings (to avoid sunlight drying of the dew), and brought to the spa located in Garniga Terme (Trentino, Italy). A 50 cm layer of grass is placed in baths and stays two or three days before naturally reaching the temperature of 55–60 °C, following spontaneous processes of self-heating. Among the most significant species constituting the grass pile, there are *Gentiana* (*pumilum, vernum, germanica, kochiana, lutea* and *verna*), *Arnica montana, Hypericum maculatum*, *Thymus serpyllum*, and *Pulsatilla alpine*^4^. By applying studied and tested techniques, it is possible to benefit from the baths also during the winter season: the collected grass is packaged and quickly brought to temperatures of − 20 °C; in this way, the thermophilic process is stopped without destroying the grass organoleptic properties.

Although PTBs are empirically known as natural treatments for rheumatic diseases^2, 5^, knowledge on the involved microbiota and investigations about the thermo-chemical process occurring in the PTB are totally missing and will help to improve the onset and the progress of the process.

This study had been essentially focused to microbiological aspects of PTB by means of both culture dependent and independent methods (partial 16S rRNA and ITS genes MiSeq Illumina sequencing). The main aim was to establish the microbial community structure at different stages of the PT process (i) identifying the bacterial and fungi community members; (ii) assessing the biodiversity of bacterial and fungal microbiota throughout the process; and finally (iii) coupling this data with the analysis of the volatiles organic compounds (VOCs) released during the process.

## Results

### Temperature and pH

**Figure 1:**
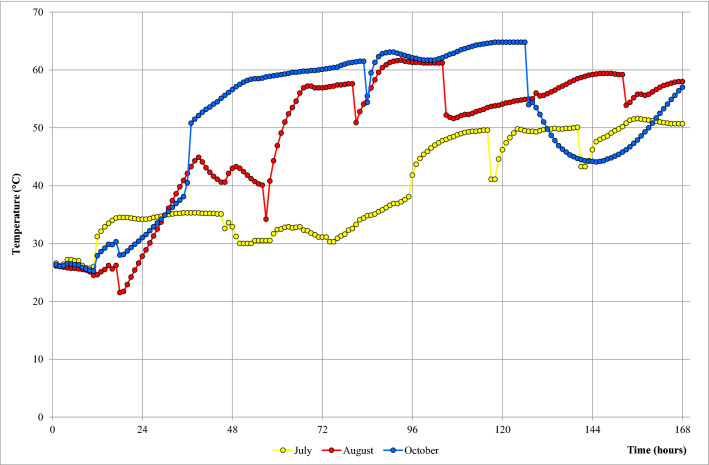
Temperature dynamic of herbs pile bath during PTB. Temperature was recorded for seven days from day 0 to d7 each hour (24 h = d1; 48 h = d2; 72 h = d3; 96 h = d4; 120 h = d5; 144 h = d6; and 168 h = d7) at 20 cm of depth in the middle of the pool bath. In yellow is the temperature trend for July, in red August and in blue October batch.

Figure 1 shows the temperature trend during the July, August and October PTBs. In the first 35 h, all the PTBs showed similar temperatures rising from 26 to 35 °C – 39 °C (Fig. 1). In the following hours, the temperature showed different trends according to the month. In July, the temperature was stable for the first four days (96 h) and then rose, reaching the max. temperature of 51 °C. In August, the temperature was stable for the first 55 h and then rose reaching the max. temperature of 61 °C. Finally, in October, after the first 35 h, the temperature rose immediately reaching 50 °C, and then was stable reaching the max. of 64.8 °C.

The pH (Table 1), in the first two days, was in a range between 6.2 and 6.5 with the exception of October when pH sowed a mean value of 7.4. After d5, the pH increased significantly and stabilized in a range between 7.4 and 7.8.

**Table 1:** Microbiological counts (Log CFU g^−1^) and pH in the herbs samples in different days and month of the PTB.

	pH	Aerobic bacteria	Mesoph. LAB	Thermoph. LAB	Entero-bacteria	Yeasts	Moulds
**July**							
day 0	6.2 ± 0.43^a^	6.7 ± 0.18^a^	n. d.^a^	n. d.^a^	3.8 ± 0.59^a^	n. d.^a^	n. d.^a^
day 2	6.2 ± 0.32^a^	7.1 ± 0.51^a^	5.3 ± 0.20^b^	2.9 ± 0.53^b^	4.6 ± 0.57^a^	n. d.^a^	n. d.^a^
day 3	6.6 ± 0.21^ab^	8.4 ± 0.29^b^	5.9 ± 0.60^b^	4.6 ± 0.52^c^	5.7 ± 1.13^b^	5.9 ± 1.36^c^	5.7 ± 0.89^c^
day 5	7.6 ± 0.26^b^	8.7 ± 0.47^b^	5.5 ± 0.74^b^	5.1 ± 1.04^c^	5.6 ± 0.67^b^	2.5 ± 1.44^b^	5.4 ± 0.59^c^
day 7	7.7 ± 0.22^b^	8.7 ± 0.31^b^	5.9 ± 0.80^b^	5.2 ± 0.87^c^	6.2 ± 0.52^b^	n. d.^a^	3.4 ± 1.20^b^
**August**							
Day 0	6.4 ± 0.31^a^	7.7 ± 0.46^a^	1.2 ± 0.80^a^	n. d.^a^	4.3 ± 0.70^a^	6.0 ± 0.64^c^	5.2 ± 0.27^b^
Day 2	6.5 ± 0.20^a^	8.1 ± 0.60 ^ab^	6.2 ± 0.57^b^	1.6 ± 0.64^a^	5.5 ± 0.99^b^	5.4 ± 1.31^c^	5.1 ± 0.47^b^
Day 3	7.0 ± 0.27^ab^	8.4 ± 0.55 ^ab^	6.1 ± 0.65^b^	4.0 ± 0.64^b^	6.5 ± 0.23^b^	3.8 ± 0.96^b^	4.3 ± 1.09^b^
Day 5	7.4 ± 0.29^ab^	8.2 ± 0.65 ^ab^	5.8 ± 1.04^b^	4.3 ± 0.84^b^	5.8 ± 0.67^b^	n. d.^a^	4.5 ± 0.87^b^
Day 7	7.6 ± 0.15^b^	8.8 ± 0.42^b^	5.9 ± 0.87^b^	5.7 ± 0.94^c^	5.7 ± 0.71^b^	n. d.^a^	3.2 ± 0.84^a^
**October**							
Day 0	7.4 ± 0.32	9.1 ± 0.46 ^b^	7.4 ± 0.80^bc^	3.8 ± 1.55^a^	5.0 ± 0.70^a^	7.8 ± 0.64	6.7 ± 0.27 ^ab^
Day 2	7.4 ± 0.74	8.9 ± 0.60 ^ab^	7.2 ± 0.57^bc^	4.5 ± 0.64^a^	5.1 ± 0.99^a^	7.7 ± 1.31	7.0 ± 0.47 ^ab^
Day 3	7.0 ± 1.10	9.4 ± 0.55 ^b^	7.6 ± 0.65^c^	5.6 ± 0.64^b^	5.9 ± 0.23^ab^	7.8 ± 0.96	7.7 ± 1.09 ^b^
Day 5	7.7 ± 0.33	8.1 ± 0.65 ^a^	6.4 ± 1.04^b^	6.2 ± 0.84^b^	5.8 ± 0.67^ab^	8.8 ± 1.48	6.6 ± 0.87 ^a^
Day 7	7.8 ± 0.24	7.6 ± 0.42 ^a^	4.9 ± 0.87^a^	5.1 ± 0.94^ab^	6.7 ± 0.71^b^	8.1 ± 0.96	6.4 ± 0.84 ^a^

Results are showed as mean values ± standard deviations of three replicates for each point of sampling (5 and 40 cm) for a total of six values. For a given column and month, microbial count or pH values with a, b and c superscripts are significantly different (*p* < 0.05).

n. d.: not detected or present in traces lower than 1.0 LOG cfu g^−1^.

### Microbial counts in herbs during PT process

The counts of viable total aerobic, mesophilic and thermophilic anaerobic bacteria, enterobacteria, yeasts and moulds in July, August and October at day 0, d2, d3, d5 and d7 are shown in Table 1. The plate counts showed no significant difference for depth of sampling (*p* value > 0.05; data not shown). The aerobic bacteria were always high at day 0 (6.7, 7.7 and 9.1 Log CFU g^−1^ in July, August and October respectively). In July and August, they significantly rose until d7, reaching similar amounts (8.7 and 8.8 Log CFU g^−1^ respectively). The mesophilic and thermophilic lactic acid bacteria (LAB) were very low in July and August at day 0 when they were present in traces or not detected (Table 1). Mesophilic LAB rose from d2 to d7 with a similar trend to aerobic bacteria reaching 5.9 Log CFU g^−1^. By contrast, in October they showed different trend: as aerobic bacteria, mesophilic LAB counts were very high at day 0 and stable without significant differences until d3 and then significantly decreased until d7 reaching 4.9 Log CFU g^−1^. Counts of thermophilic LAB showed similar trends in all the three months: they rose until d7 reaching similar amount in July, August and October (5.2, 5.7 and 5.1 Log CFU g^−1^ respectively). Enterobacteria counts were lower in herbs at day 0 (3.8, 4.3 and 5.0 Log CFU g^−1^ in July, August and October respectively) and then significantly rose until d7 reaching 6.2, 5.7 and 6.7 Log CFU g^−1^ in July, August and October respectively. In July, yeasts and moulds in the first two days were not detected or present in traces, then reached their highest value at d3 and significantly decreased until d7. By contrast, their counts were very high in both August and October; in particular, moulds counts trends were similar: they significantly decreased until d7 to 3.2 and 6.4 Log CFU g^−1^ in August and October respectively.

### Characteristics of the sequencing data

The DNA extracted from the 90 PTB samples had been all successfully amplified. After merging and quality trimming the raw data, 2,980,511 reads for bacteria and 1,227,092 reads for fungi remained for subsequent analysis (Table S1). After alignment, the remaining Operational Taxonomy Units (OTU) had been clustered at a 3% of distance.

### Bacteria and fungi: alpha diversity

The number of OTUs and the Shannon diversity index were determined using QIIME2 at 97% similarity levels (Table 2), in order to analyse the bacterial and fungi community richness in samples obtained during the PT process. Regarding the sample position (5 and 40 cm depth), there was no significant difference in both observed OTUs number and Shannon diversity index for bacteria and fungi communities (*p* value > 0.05). It is worth noting that the degree of bacterial diversity was significantly higher in July and August than in October samples, by contrast, the degree of fungal diversity was significantly higher in July and October than in August samples (Shannon diversity index in Table 2).

**Table 2:** Observed OTUs (Obs. OTUs) and Shannon diversity index (Shannon div. ind.) in the herbs at different depth, month and of day sampling of the PTB.

	Bacteria		Fungi	
	Obs. OTUs	Shannon div. ind.	Obs. OTUs	Shannon div. ind.
**Depth of sampling**				
5 cm	340 ± 126	6.9 ± 1.19	82 ± 48	3.7 ± 1.75
40 cm	259 ± 87	6.5 ± 1.15	70 ± 56	3.3 ± 2.02
**Month of sampling**				
July	381 ± 136^a^	7.4 ± 0.76^a^	85 ± 56	3.6 ± 1.00^ab^
August	369 ± 289^a^	7.2 ± 0.77^a^	59 ± 39	2.6 ± 2.09^a^
October	153 ± 111^b^	5.5 ± 1.17^b^	85 ± 38	4.2 ± 1.21^b^
**Day of sampling**				
Day 0	318 ± 166^a^	7.0 ± 1.14^a^	133 ± 16^a^	5.3 ± 0.35^a^
Day 2	451 ± 268^a^	7.1 ± 1.56^a^	106 ± 38^a^	4.6 ± 1.17^a^
Day 3	411 ± 209^a^	7.1 ± 0.98^a^	77 ± 49^b^	3.3 ± 1.87^b^
Day 5	177 ± 96^b^	6.2 ± 1.13^b^	41 ± 35^bc^	2.6 ± 1.71^b^
Day 7	138 ± 28^b^	6.0 ± 0.62^b^	22 ± 19^c^	1.6 ± 1.11^c^

Results are showed as mean values ± standard deviations.

For a given column and month, observed OTUs and Shannon diversity index values with a, b and c superscripts are significantly different (*p* < 0.05).

The variation in number of OTUs and Shannon diversity index over time indicates highest microbial diversity at day 0 and d2, and a lower microbial diversity at d5 and d7.

### Bacteria and fungi: beta diversity

**Figure 2:**
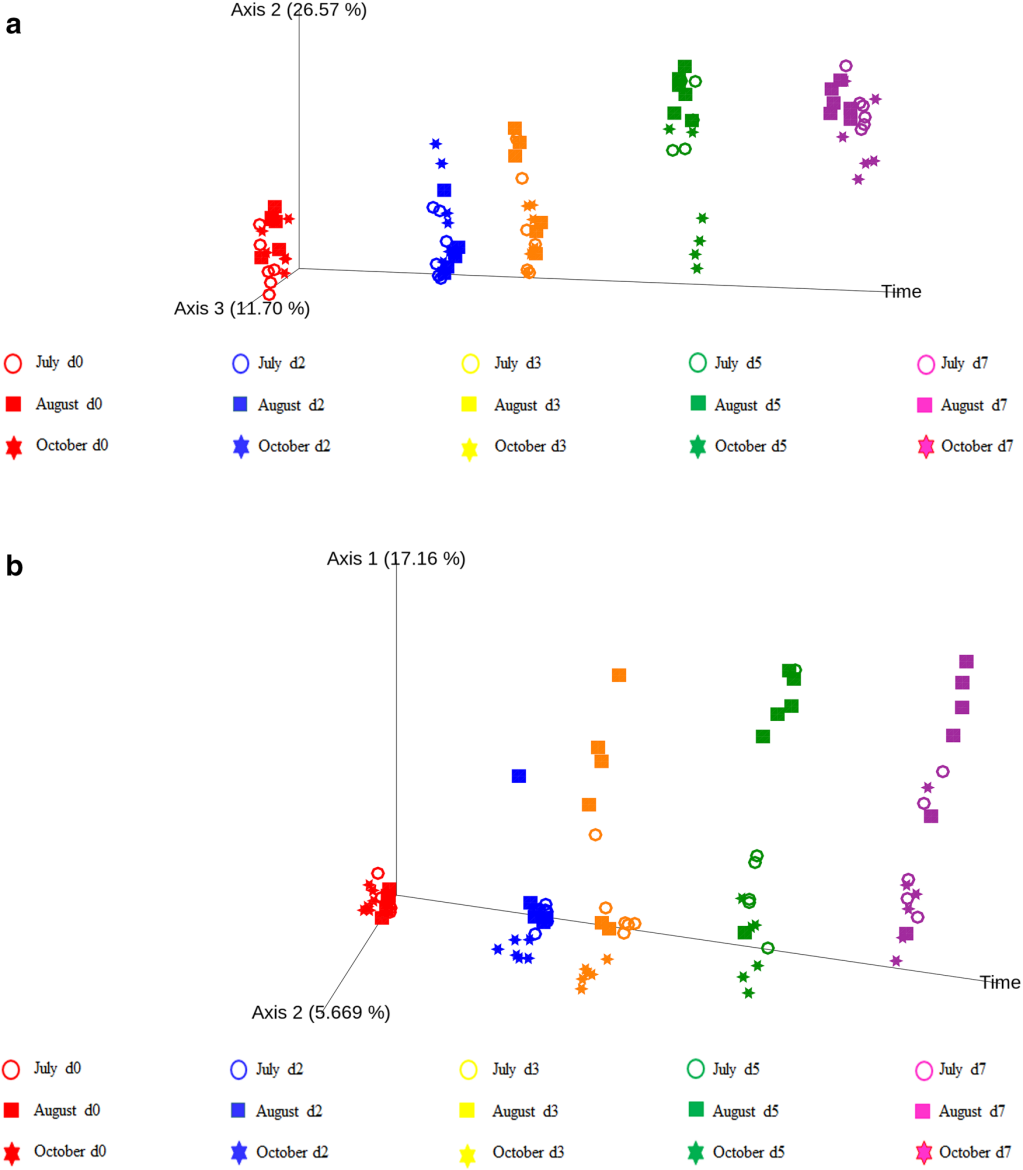
Principal coordinate analysis of Weighted UniFrac distances for bacterial community (**a**) and Bray–Curtis distances for fungi community (**b**) in PTB. The Time custom axis has been used to show the PCoA changes in the days. For interpretation of the symbols and colors the reader is referred to the legend.

The PCoA of UniFrac and Jaccard metric indicated clear clustering of both bacterial and fungi communities according to the different PTB days (Fig. 2a,b).

Bacterial and fungi communities were more phylogenetically dissimilar between successional days (day 0, d2, d3, d5 and d7) or months (July, August and October) than between position of sampling (5 and 40 cm). The bacterial Weighted UniFrac PCoA (Fig. 2a, total variation explained: 52.09%) and the fungal Jaccards PCoA (Fig. 2b, total variation explained: 32.83%) revealed a clearer picture of the similarities across different days. The PCoA plots emphasized the similarities of the bacterial and fungi communities in the PTB at d2 and d3 when compared with d5 and d7.

These results were supported by the PERMANOVA statistical analysis (Table 3). The differences between position of sampling (5 and 40 cm) were not significant both for bacteria and fungi communities as the *p* values were 0.19 for bacteria and 0.27 for fungi community respectively. The differences among months of sampling were significant comparing July and August with October for both bacteria and fungi communities as well as the differences among microbial communities through time. The pairwise comparison (Table 3) clearly showed two significantly different stages during the PTB after day 0: the first stage including d2 and d3 (1st stage), and the second stage including d5 and d7 (2nd stage). The time effect on microbial composition (pseudo-F value) was showing a growing trend with the proceeding of the sampling days (Table 3). The bacterial pseudo-F values were smaller and smaller with progression of the days; by contrast, fungi pseudo-F values were higher. This means that the bacteria became more similar and the fungi more different in the samples from the three piles studied as the process progressed.

**Table 3:** Permanova analysis (999 permutations) results for bacterial and fungi communities based on weighted unifrac and Jaccard distances respectively.

	Bacteria		Fungi	
	Pseudo-F	*p* value	Pseudo-F	*p* value
**Main effects**				
Depth of sampling	2.822	0.29	1.612	0.23
Month of sampling	4.447	0.001**	3.600	0.001**
Day of sampling	10.553	0.001**	3.348	0.001**
**Pairwise comparisons**				
July versus August	2.177	0.071	2.495	0.002**
July versus October	4.815	0.003**	4.485	0.001**
August versus October	6.253	0.003**	3.834	0.001**
**Pairwise comparisons**				
Day 0 versus day 2	11.033	0.001**	1.362	0.015*
Day 0 versus day 3	13.459	0.001**	2.633	0.001**
Day 0 versus day 5	17.984	0.001**	5.538	0.001**
Day 0 versus day 7	18.172	0.001**	7.545	0.001**
Day 2 versus day 3	0.808	0.495	1.349	0.096
Day 2 versus day 5	7.957	0.001**	3.630	0.001**
Day 2 versus day 7	15.168	0.001**	5.556	0.001**
Day 3 versus day 5	5.392	0.002**	1.825	0.025*
Day 3 versus day 7	12.266	0.001**	3.187	0.001**
Day 5 versus day 7	3.421	0.12	1.097	0.232

Significance levels: *p < 0.05; **p < 0.01.

### Bacterial community structure

**Figure 3:**
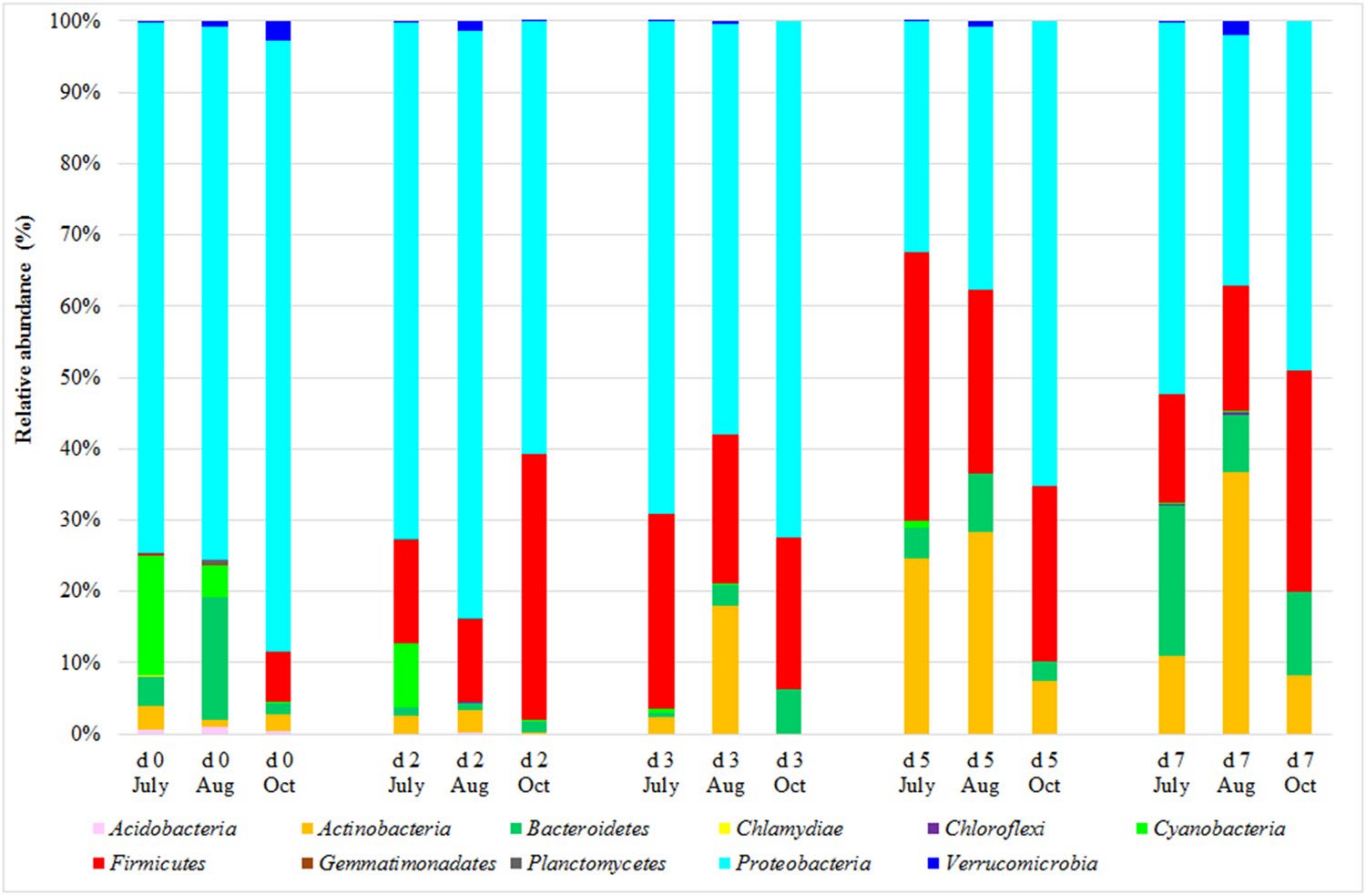
Phylum composition (in mean relative abundance) of herbs samples as revealed by high-throughput sequencing analysis. The samples were collected in triplicate from the same pool, for five days (day 0, d2, d3, d5, and d7) at 5 and 40 cm of depth, in July, August (Aug) and October (Oct). For interpretation of the references to color in this figure legend, the reader is referred to the web version of this article.

**Figure 4:**
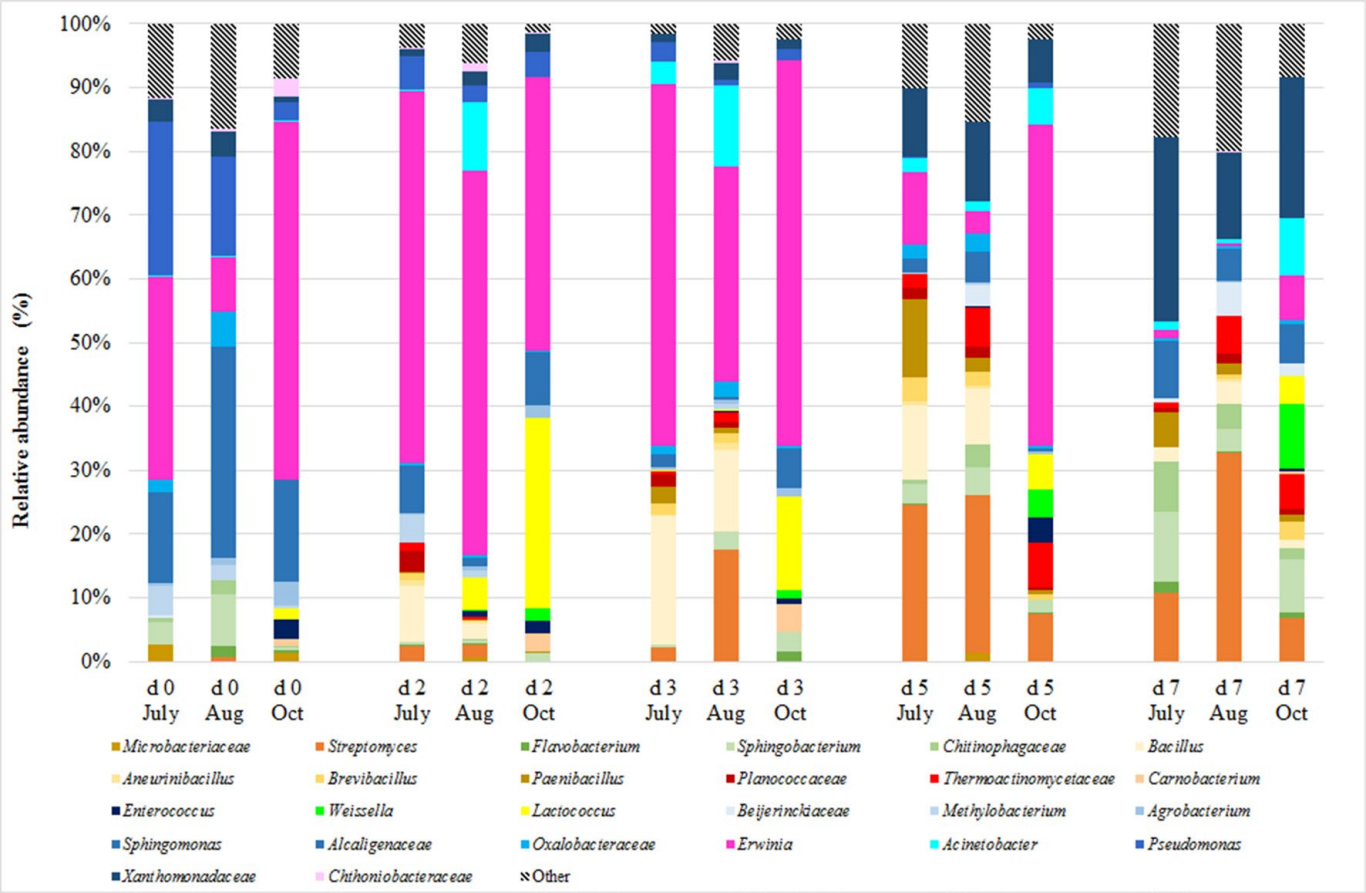
Bacterial taxa groups (genus level or above) composition, in mean relative abundance, of herb samples as revealed by high-throughput sequencing analysis. The samples have been collected in triplicate from the same pool, for five days (d0, d2, d3, d5, and d7) at 5 and 40 cm of depth, in July, August (Aug) and October (Oct). (For interpretation of the references to colour in this figure legend, the reader is referred to the web version of this article).

Eleven prokaryotic phyla were found in the 90 samples accounting for the total bacterial community. The predominant phylum across all bacterial communities was the *Proteobacteria*, accounting for 33%–83% of the OTUs in each time and month of the PTB (Fig. 3). The trend of *Proteobacteria* was similar in all the months with different amount of presence: at day 0 and d2 *Proteobacteria* abundance was between 61% and 85%; at d3, showed a slight decrease (between 58% and 72%) and reached the lower values at d5 and d7 (between 33% and 65%). Twenty-six bacterial phylotypes were found dominant across all samples, accounting for the 90% of the total bacterial community. Of these 26 phylotypes (in Fig. 4), 10 belonged to *Proteobacteria*. *Erwinia* was the most abundant genus in this phylum and reached the higher presence at d2 and d3. Other frequently sequenced genera included *Acinetobacter*, *Pseudomonas*, mainly found at day 0, and unclassified genera belonging to *Xanthomonadaceae* family.

Of the *Alphaproteobacteria*, the genera *Agrobacterium*, *Methylobacterium*, *Sphingomonas* and unclassified genera of the family *Beijerinckiaceae* were the most abundant and dominant at day 0. All these phylotypes showed a lowering trend in time decreasing from day 0 to d7 with the only exceptions of the *Beijerinckiaceae* that were higher at d7 than at day 0.

Of the *Betaproteobacteria,* unclassified genera belonging to *Alcaligenaceae* and *Oxalobacteraceae* were the most abundant families. Their relative abundances were constant along the time and showed higher differences among the months than the sampling days (average values of relative abundance were 2.4%, 4.5% and 0.5% in July, August and October respectively).

*Firmicutes* accounted for 12%–38% of the OTUs from d2 to d7 of the PT process. At day 0, *Firmicutes* were present only in October’s samples at 7%, then increased at d2 and remained stable until the end of the process (Fig. 3). Overall, there were 10 *Firmicutes* in the 26 most abundant phylotypes found during the PTBs (Fig. 4). Most of these were thermophilic bacteria such as *Bacillus*, *Thermoactinomycetaceae*, *Brevibacillus* and *Paenibacillus*^6^. These themophilic phylotypes had never been detected at day 0, appeared at d2 and reached their higher values at d5 when their presence accounted for 29.9%, 19.4% and 8.5% in July, August and October respectively.

The *Bacteroidetes* constituted another dominant phylum detected in all the samples (Fig. 3). *Bacteroidetes* abundance at d0 was in the range of 1.4%–17.0%, decreased at d2, d3 and d5 to a range of 0.7%–8.1% and reached the highest values at d7 (between 8.1% and 21.2%). The most abundant genera belonging to this phylum were *Flavobacterium* and *Sphingobacterium*.

The abundance of *Actinobacteria* was 1.0%–3.3% at day 0 and increased until the end of the process reaching a maximum of 7.5–36.6% relative abundances respectively (Fig. 3). The most abundant phylotypes in the *Actinobacteria* phylum were *Microbacteriaceae* and *Streptomyces* (Fig. 4); in particular, *Streptomyces* was one of the genera dominating bacterial community biodiversity at d5 and d7.

*Cyanobacteria* were found at relative abundances higher than 1% only in July and August samples at day 0 and d2, with a maximum at day 0 (16.9% and 4.5% relative abundances in June and July samples respectively). After d2, Cyanobacteria decreased until the end of the process (Fig. 3). The relative abundance of *Verrucomicrobia* was 0.35%–2.8% at d0 and remained constant during the whole PTB process (Fig. 3). *Chtoniobacteraceae* was the most abundant bacterial family of the *Verrucomicrobia* phylum.

Further bacterial phyla had always been found at very low relative abundances (never higher than 1.0%, Fig. 3).

### Fungal community structure

**Figure 5:**
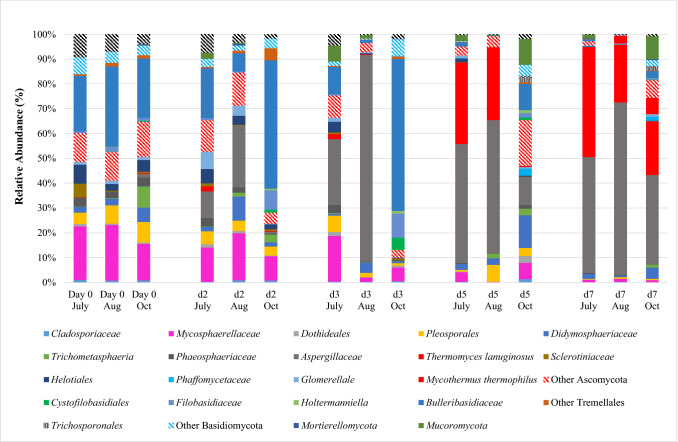
Fungi taxa groups (genus level or above) composition, in mean relative abundance, of herb samples as revealed by high-throughput sequencing analysis. The samples were collected in triplicate from the same pool, for five days (d0, d2, d3, d5, and d7) at 5 and 40 cm of depth, in July, August (Aug) and October (Oct). (For interpretation of the references to color in this figure legend, the reader is referred to the web version of this article).

Before the PTB started, the fungal community in the herbs (day 0 in Fig. 5), was dominated by *Mycosphaerellaceaes* (*Mycosphaerella, Ramularia* and *Zymoseptoria* genera), representing the 21.5%, 21.4% and 15.0% of the total in July, August and October respectively, and *Bulleribasidiaceaes* (*Vishniacozyma,* and *Dioszegia* genera) representing the 22.3%, 32.4% and 23.4% of the total in July, August and October respectively. Other fungal taxonomic groups, mainly belonging to the *Ascomycota* phylum, were detected in lower relative abundance (lower than 10%). After two days (d2), the *Aspergillaceae* family was emerging, mainly constituted by the *Aspergillus* genus with traces of *Penicillium* in 13 out of the 90 samples. *Aspergillaceae* dramatically increased from day 0 (always less than 1%) to d2 in July and August trials (10.6% and 24.9% respectively), and after d3 they became the most dominant fungi (26.6% and 83.2% respectively). By October, *Bullerobasidiaceae* was always the dominant fungal family at d2 and d3. After five days (d5 in Fig. 5), *Bulleribasidiaceaes* (lower than 10.6%) and *Mycosphaerellaceae* (lower than 6.5%) relative abundances decreased sharply. *Aspergillaceae* kept their rising trend, remaining the dominant fungal family in July and August trials (47.9% and 56.6% respectively), but they represented only the 11.4% of the fungal relative abundance in October. Other thermophilic species emerged in July and August: the *Trichocomaceae* mainly constituted by *Thermomyces lanuginosus* whose relative abundance was never higher than 2.1% in the first three days and then suddenly increased to 32.8% and 23.2% in July and August respectively.

After seven days (d7 in Fig. 5), the fungal community was totally dominated by *Aspergillaceae* (46.6%, 69.2% and 35.9% in July, August and October respectively) and *Thermomyces lanuginosus* (44.2%, 23.3% and 21.6% in July, August and October respectively). None of the OTUs was predominant throughout all samples.

### Volatiles organic compounds (VOCs) released during the PTBs

**Figure 6:**
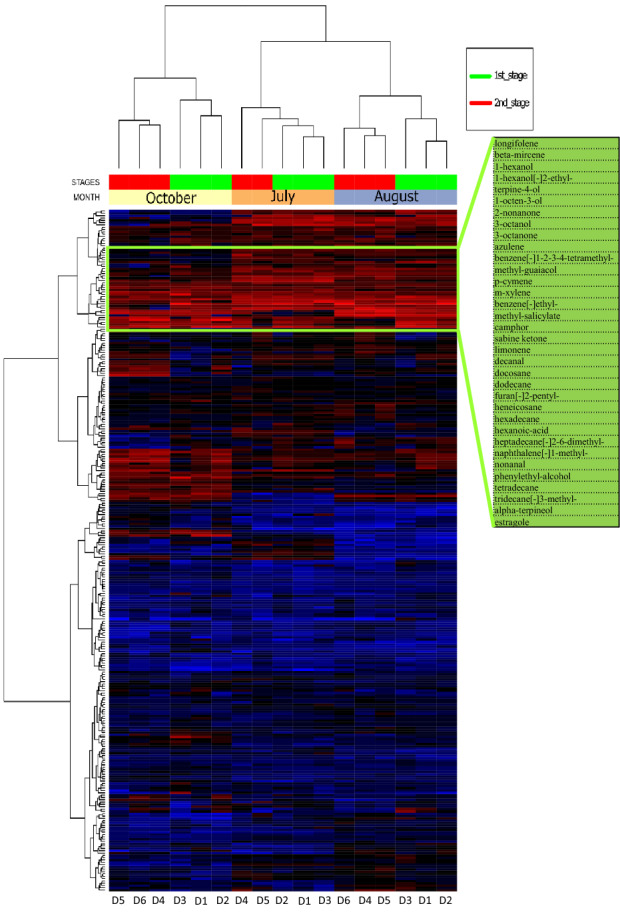
Heatmap and hierarchical clustering based on the normalized quantities of the identified VOCs, for PTB herbs in the six days (d1, d2, d3, d4, d5, and d6) and three months (July, August and October) of sampling. The highest content is in red and the lowest in blue. The values have been UV scaled and clustered according the Ward algorithm. The list of the 34 compounds highlighted in the upper side represents the most abundant (core) VOCs found. In July, d6 is missing as the sample has not been collected.

After raw GCxGC–MS data deconvolution and pre-processing, the three dataset (July, August and October) consisted of 722, 1105 and 815 volatiles respectively. As already reported by Narduzzi et al.^7^, the majority of the VOCs are not in common among the months. The identified volatiles through all the three months’ datasets were matched using their InchiKey, and produced a table consisting of 295 VOCs present in all the months (Table S2). As shown in the top part of the Fig. 6, there is a cluster of 34 compounds that are the most representative of all PTB samples because contributing to the 85% of the total VOCs mass emissions. In the heatmap Fig. 6, the samples from the same month clustered together. Moreover, within each month, the samples split in two different clusters according to the stages already identified in the microbial analysis. The first cluster is composed by the samples of d1, d2 and d3 (1st stage) and the second cluster by the samples of d4, d5, and d6 (2nd stage). Looking at the differences in the days within each batch, the d1, d2 and d3 samples were richer in aliphatic hydrocarbons (heneicosane, hexadecane, tetradecane and 3-methyltridecane), alpha-terpineol, and estragole. By contrast, the d4, d5 and d6 samples were richer in 1-methylnaphthalene, nonanal, 2-nonanone, 3-octanol, m-xylene, 2,6-dimethylheptadecane and 2-ethyl-hexanol.

## Discussion

The PTB is documented as a natural healthy treatment for human rheumatic diseases^2, 5^, but this is the first study investigating the microbiota and VOCs occurring in this process. The only similar processes are the self-heating ones occurring during composting^8, 9^ or silage fermentation^10^. In order to establish the different stages of PTB, the temperature, pH, microbial composition, microbial abundance, and VOCs data had been recorded for seven days in three different months: July, August, and October.

The PTB temperature was always increasing from ambient temperature (25 °C) until more than 50 °C with great variation among the different months, in particular the temperature rise over 50 °C, was very fast in August and October (never longer than 2.5 days). This trend is very similar to other self-heating processes where the initial mesophilic phase (until 42 °C) may last for only a few hours or a couple of days^8^. The different temperature trends among the three months suggested heterogeneity of the PTB that could reflect differences in herbs composition, compaction and aeration of the pile as well as in the active microorganism populations. The day-by-day addition of the bulking herbs determined an aeration and regeneration effect within the PTB pile, increasing substrate and oxygen availability and likely determining a boosting effect on the microbial growth and the heating process^11^.

In general, the pH remained in a neutral range in all the PTBs. In July and August, after d3, it increased significantly and then stabilized in a range between 7.4 and 7.8 that is suitable for most microorganisms’ life^8, 9^. No pH drop was recorded, contrary to what occurring in further self-heating processes where microorganisms rapidly break down soluble and easily degradable carbon sources, resulting in a pH drop from 6.5 to 6.0 due to organic acids accumulation^8^. This is in agreement with bacteria and fungi counts that were two orders of magnitude lower than in other self-heating processes^8^ and with the volatile acids data that where not among the core of the recorded VOCs. Only in October, the pH showed a (non-significant) drop, between d2 and d3. It was probably caused by the generation of organic acids from *Aspergillaceae* species^11^ that showed high relative abundance (Fig. 5) and counts (Table 1, moulds counts) already after three sampling days in October.

Regarding microbial counts, bacteria and fungi showed lower counts (at least two orders of magnitudes) than microbial population of other self-heating processes^8^, with the exception of October when microbial counts were at higher amount. In general, aerobic bacteria dominated the PTB microbiota (around three orders of magnitude higher than other microbial groups). Therefore, aerobic bacteria were the main actors leading the PTB self-heating, probably by means of an aerobic exothermic step already known as a bio-oxidative process typical of the first phase of the composting process^12^. According to our results, the microbial evolution throughout the PTB was a very dynamic process, and the temperature did not result as deleterious for bacteria as reported for other self-heating process^12^. Microorganisms’ kept growing albeit temperature reached values higher than 50 °C, showing that most of them were thermo-tolerant.

Fungi counts showed a different trend when compared with bacteria. In July and August, the yeasts and moulds showed always very low counts following a significant decreasing trend at d5 and d7, similar to the one reported by Woolford^10^ in silage fermentation. This decrease is probably due to the difficult adaptation to the high temperatures. Therefore, temperature could be a selective factor for fungal community in PTBs, similarly to the results of Zhang et al.^13^, that reported high temperature as the driving factor affecting the fungal population in waste pile composting.

Alpha and beta diversities were analysed to study the bacterial and fungi community richness in samples obtained during the seven days of the PTB process. The first 3 days showed the highest bacterial and fungal alpha-diversity. After d5, the bacterial and fungal diversity significantly decreased which could be due to a high competitive selection, occurring in environments with a high nutrient complexity containing similar species competing for the same resources^14^. The depletion of organic substrates could lead to a gradual disappear of less competitive taxa and to a microbial community convergence towards stable composition of limited diversity^14^. Both the OTUs number and Shannon diversity index of fungi showed a significant constant lowering trend indicating that fungal diversity decreased with temperature rise, confirming that the high temperature had a certain degree of selectivity against fungi.

All these results suggested the existence of two-stages in the PTB process after day 0. The 1st stage was characterised by temperature boosting until d3, higher microbial diversity and a predominance of the bacterial development among the microbial community, and the 2nd stage, from d5 to d7, characterised by almost constant high temperature, a minor bacterial and fungi diversity and higher pH than 1st stage.

The presence of a two-stage process has been clearly confirmed also by the beta-diversity. The microbial communities in the first three days showed significant differences when compared to the last two days of the PT process. No significant difference had been detected at sampling positions 5 and 40 cm suggesting a certain homogeneity in the pool of the PTB that makes the samples representative of the overall complete batch whatever the deep of sampling. Although the succession of microbial communities occurred with some differences in July, August and October, the overall microbial community was similar according to the PTB stage in all the months of sampling. This suggests that there is a similar bacterial and fungal phylogenetic turnover pattern during the PTB processes, alongside a comparable progression. The significant differences among the microbial communities of July, August and October could be associated to differences in herb composition, compaction and aeration of the pile that leads to differences in microbial composition and temperature trend.

Analysing the structure of the bacterial community developing during the PTB process, the predominant bacteria were belonging to *Proteobacteria* phylum and, in particular, *Erwinia* was the most abundant genus in the 1st stage. This genus is known to include phyto-pathogenic bacteria and it was never found in previous study about self-heating microbial processes^8–10^, confirming the peculiarity of PTBs. *Acinetobacter*, *Pseudomonas* and *Xanthomonadaceae* were also bacterial phylotypes belonging to *Proteobacteria* phylum whose sequences were abundant in the PTBs samples. *Acinetobacter* and *Pseudomonas* are generally recognised as mesophilic genera and probably this explain their higher abundance in the 1st stage and the drop following the temperature increase, as reported in others self-heating processes^8, 9^. Bacteria belonging to *Xanthomonadaceae* family can live both in thermophilic and extremely thermophilic environments^9^, and probably for this reason, they were dominant in the 2nd stage of the PT process when temperatures were always over 50 °C. The *Firmicutes* relative abundance, mainly represented by *Bacillales* (*Bacillus* and *Thermoactynomycetaceae*), increased after two days and then remained a constant presence in the 2nd stage, similarly to Partanen et al.^15^ that showed *Bacillales* prevalence during the thermophilic phase of self-heating composting processes. *Bacteroidetes* constituted another phylum detected in all the samples. The most abundant genera belonging to *Bacteroidetes* were *Flavobacterium* and *Sphingobacterium,* present along the overall PTB process without differences between 1st and 2nd stages. *Flavobacterium* and *Sphingobacterium* are known as active bacteria during the composting of lignocellulosic materials^16, 17^. The most abundant phylotypes belonging to *Actinobacteria* phylum were *Microbacteriaceae* and *Streptomyces*. In particular *Streptomyces* abundance was higher in the 2nd stage in agreement with previous studies that found this species predominant during the thermophilic stage of self-heating organic compounds processes^16, 18^ as they can tolerate high temperatures and are able to degrade cellulose and lignin^8, 15, 19^.

Analysing the structure of the fungal community developed during the PTB process, the dominant groups, before the temperature rises, were *Mycosphaerellaceaes* and *Bulleribasidiaceaes* families. *Mycosphaerellaceae* is one of the largest family of *Ascomycota* and comprises many important crop pathogens^20^. *Bulleribasidiaceae* belong to *Basidiomiycota* phylum and is constituted by well cold-adapted genera recovered in cold terrestrial areas like Himalayas, Andes, and European high mountains^21, 22^; their cold adaptation could explain why they were found in very high amount at day 0 in the fresh-cut grass from Viote area where the average year temperature is about 5.5 °C^23^. After two days, a new fungal family emerged when the temperatures rises: the *Aspergillaceae*, a family able to resist high temperatures^24^ and known to be one of the dominant fungi families in self-heating processes^8^. After three days, other thermophilic species emerged to become dominant in the 2nd stage like *Thermomyces lanuginosus,* whose presence in thermophilic stage of self-heating process was already documented^8^. *Aspergillaceae* and *Thermomyces lanuginosus* predominance in the 2nd stage was probably due to their thermophilic attitude and their potential in producing thermostable enzymes^25, 26^.

The majority of the VOCs released during PTB process were not in common from among the months, confirming the heterogeneity of the herbs constituting the PTBs. The different concentrations and the presence of unique VOCs in the three months of analysis might be due, not only to different herbs and microbial populations in the collected samples, but also to the competitive binding of the volatiles on the used sampling tubes, limiting the repeatability of VOCs quantification. The VOCs analysis revealed the presence of 34 compounds constituting the core of the most abundant compounds in the PTB process. Most of them were monoterpenes and sesquiterpenes such as longifolene, myrcene, camphor, limonene, terpinen-4-ol and cymene. They were constantly present in all the samples and are known to be the major chemical compounds of plant essential oils^27, 28^. They have been reported to possess a wide range of bacterial inhibitory potential in particular against pathogens like *Escherichia* and *Staphylococcus* genera^27^ and terpinen-4-ol in particular can be used in the therapy of skin infections^29^. The antibacterial activity of these compounds may justify the lower counts found both for bacteria and fungi when compared with generic self-heating processes^8^, and the absence in the PTBs of pathogenic bacterial genera, such as *Escherichia*, *Staphylococcus, Serratia, Enterobacter* and *Klebsiella*, that are very common in self-heating composting process^8^.

Considering the 1st stage of the PTB process, there was a higher concentration of aliphatic hydrocarbons (heneicosane, hexadecane, tetradecane and 3-methyltridecane), alpha-terpineol, and estragole than at the 2nd stage. By contrast, the 2nd stage was richer in 1-methylnaphthalene, nonanal, 2-nonanone, 3-octanol, m-xylene and 2,6-dimethylheptadecane. The compounds more representative of the 1st stage of PTB process were of plant origin, because common component of the volatile oils of the herbs and flowers constituting the PTB pile and in some cases have been shown to have significant health effects. Aliphatic hydrocarbons are constituents of *Arnica montana* buds^28^ and *Hypericum maculatum* flowers^30^. Estragole is a natural anise odor component of plant essential oils and has in vivo anti-inflammatory action^31^. Natural alpha-terpineol is a lilac odor compound that can be produced by fungal (*Penicillum* sp.) or bacterial (*Pseudomonas* sp.) biotransformation of limonene^32^ and has antioxidant and anti-proliferative potential^33^. Regarding the 2nd stage of PTB process, with the exception of nonanal that is one of the main constituents of *Arnica montana* buds^28^, the most representative compounds were of microbial origin and had been recorded among *Aspergillum* volatile metabolites such as 1-methylnaphthalene, 3-octanol, xylene and 2,6-dimethylheptadecane^34^, and *Bacillus* volatile metabolites such as 2-nonanone^35^.

In conclusion, this is the first study about the PTB process, which is a traditional treatment for rheumatic diseases widespread in some Alpine areas, but, due to its peculiarity and limited area of diffusion, has never been previously studied. The PTB process has some similarities with compost and silage self-heating processes such as: (i) the rapid rise in temperature over 50 °C, (ii) the presence of two stages, one more mesophilic and one more thermophilic, (iii) the rise in pH from 6.5 to about 7.5, (iv) the development of some microbial species in the 2nd stage, such as *Streptomyces*, *Xanthomonadaceae*, *Aspergillaceae* and *Thermomyces*. More relevant are the differences that confirm the originality of the PTB process when compared with compost and silage self-heating processes such as: (i) the lower counts of both bacteria and fungi, (ii) the absence of pathogenic bacteria, (iii) the dominance of *Erwinia* sp. in the 1st stage which could be the main actor of self-heating in PTB process, (iv) the dominance of plant terpenoids among the VOCs, some of them with health characteristics^27, 29^, (v) a 1st mesophilic stage characterized by plant VOCs with some beneficial effects^27, 29, 31^, and (vi) a 2nd thermophilic stage characterized by the *Aspergillaceae* presence and the production of VOCs of microbial origin.

Nevertheless, it is worth noting that there are some fungi, such as *Aspergillaceae,* that could be pathogenic to humans in particular within the 2nd stage of the PTB process and could be a real problem for operators and customers^36^.

Therefore, additional studies should be conducted to evaluate the possibility of extending the 1st stage in order to lower the development of *Aspergillaceae* and to promote the extended release of plant VOCs with beneficial characteristics. The extension of the 1st stage could be achieved setting up a system of better temperature and aeration control, also increasing the amount of bulk herb added each day (for instance from 10% to 20%). The pH and temperature record could be a direct measure to check the transition from the first to the second stage because both values are easy to detect and increase significantly between the two stages.

Based on the results of this investigation, the authors strongly recommend taking action to reduce the workers’ exposure to *Aspergillaceae* and harmful fungi that could be raised during total removal of the herbs at the end of the process.

## Methods

### The phyto-thermal bath (PTB)

**Figure 7:**
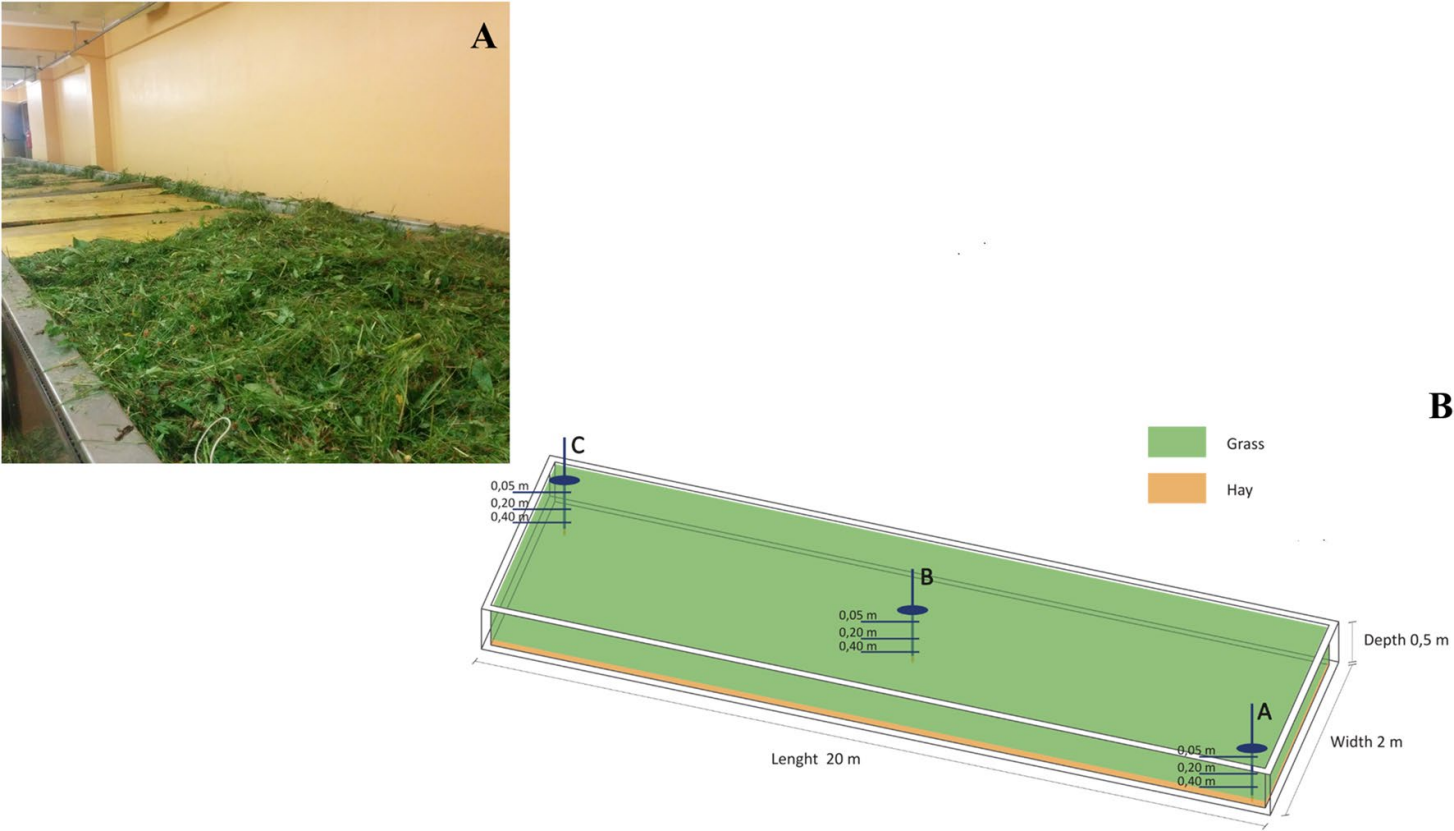
(**A**) Picture of the grass stockpile at the beginning of the PTB with a (**B**) schematic representation of the grass bath.

In order to study the real PTB process, traditional PTB has been performed in the original facility bath at Garniga Terme Spa, following the original protocol. The grass was harvested on July and August 2016, in Viote, (Trento, Italy, N 46°01′16″; E 11°02′11″), elevation 1540 m above sea level, sandy loam soil type, with average year precipitation of 576 mm/m^2^, and average year temperature of 5.5 °C^24^. The grass had been mowed and directly baled to be transported to the thermal facility of Garniga (Trento, Italy, S 23°38′57″; W 46°37′19″) where the PTB process was carried out following the protocol described by Narduzzi et al.^7^. The facility has four 20 m^3^ stainless steel open baths (Fig. 7) and is designed to host about 1 ton of grass for each cycle in each bath. The herbs had been layered in the baths to create a 45 cm tall pile, and covered with wooden bars. Adequate aeration of the pile had been kept by daily adding a 10% of bulk herbs, turning every day the herbs through a dedicated machine. After seven days, the PTB process within the baths had been considered finished and the material removed.

### Sample collection

About 50 g samples had been collected throughout the PTB process from the pool in three different months: July, August and October. After completion of the PTB pile, samples were collected at the arrival of the herbs at the facility (day 0) and then at d2, d3, d5 and d7 of each trial. To obtain a representative sample of the cell, for each sampling day, the pile was sampled in three different points, along the cell diagonal: two edges (A and C Fig. 7B) and in the middle (B in Fig. 7B), at 5 and 40 cm depths. Each sample was stored into two sterile 50 mL Falcon tube. One tube was immediately frozen in liquid nitrogen and stored at − 80 °C until DNA extraction. The second tube was kept at 4 °C and used for the immediate pH and microbial counts analyses.

### Temperatures and pH determination

Temperature had been recorded for seven days from day 0 to d7, each hour at 20 cm in the middle of the pool bath (0.20 m point B in Fig. 7B). The temperature of the room and of the PTB pile has been determined using a Testo 175 T2 (Testo limited, Alton, UK). The pH was determined from suspensions of fresh grass samples in 0.9% sodium chloride using a pH electrode (Crison Instruments, Barcelona, Spain).

### Microbial analyses

All the herbs samples were subjected to microbial analysis and prepared by adding 180 g of sterile (autoclaved for 15 min at 121 °C) peptone water to 20 g of sample. The sample was run twice for 60 s at the setting “normal speed” in a laboratory blender (Stomacher Seward 3500; Worthing, West Sussex, UK). Since this was the first work on PTBs’ microbial counts, it was not clear which kind of viable microbial groups we could find nor their respective abundance. In order to acquire more information, several microbial groups (mesophilic and psychrotrophic aerobic bacteria, mesophilic and thermophilic lactic acid bacteria, Enterobacteria, Acetobacteria, yeasts and moulds) were determined on ten subsamples as a preliminary experiment. Ten decimal dilution series were made and all plated, in order to determine the microbial groups present and the order of magnitude. Enumeration of the present microbial groups (total aerobic bacteria, mesophilic and thermophilic lactic acid bacteria, Enterobacteria, yeasts and moulds) were made from the last four decimal dilutions where the microbial group had shown to be present according the results from the preliminary experiment. Total bacteria had been cultured aerobically on Plate Count Agar (PCA) at 30 °C for 24 h. Lactic acid bacteria (LAB) were cultivated under anaerobic conditions on MRS agar plates for 72 h at 30 °C for mesophilic and 45 °C for thermophilic bacteria. Enterobacteria had been cultured on violet red bile glucose agar (VRBGA) following the overlay method at 37 °C for 24 h. Fungi had been cultured on WL agar plates, and aerobically incubated at 25 °C. Yeasts and moulds had been counted after two and five days respectively. All media had been purchased by Oxoid (Oxoid Ltd, Cambridge, UK).

### DNA extraction

All the samples collected, had been subjected to DNA extraction, performed with MoBio DNA Power Soil kit (Mo Bio Laboratories Inc., Qiagen Venlo, Netherlands) following vendor’s instructions. All DNA samples were purified by PowerClean DNA Clean-up Kit (Mo Bio Laboratories Inc.) and quantified by Nanodrop3300 Fluorospectrometer (Thermo Scientific, Waltham, USA) using the Quant-iT PicoGreen dsDNA Assay Kit (Invitrogen Life Technology, Thermo Scientific).

### Miseq library preparation and Illumina sequencing

Amplicon library preparation, quality and quantification of pooled libraries were performed at the Sequencing Platform, Fondazione Edmund Mach (FEM, San Michele a/Adige, Italy). Briefly, for each sample, a 464-nucleotide sequence of the V3-V4 region^37, 38^, of the 16S rRNA gene (*Escherichia coli* positions 341 to 805) and ITS1F (5′-GTTTCCGTAGGTGAACCTGC-3′) and ITS4 (5′-TCCTCCGCTTATTGATATGC-3′) specific for the ITS1-5.8S fungi region^39^ were amplified for bacteria and fungi respectively. Unique barcodes were attached before the forward primers to facilitate the pooling and subsequent differentiation of samples. To prevent preferential sequencing of the smaller amplicons, the amplicons were cleaned using the Agencourt AMPure kit (Beckman coulter, Brea, USA) according to the manufacturer’s instructions; subsequently, DNA concentrations of the amplicons were determined using the Quant-iT PicoGreen dsDNA kit (Invitrogen Life Technology) following the manufacturer’s instructions. In order to ensure the absence of primer dimers and to assay the purity, the generated amplicon libraries quality was evaluated by a Bioanalyzer 2100 (Agilent, Palo Alto, CA, USA) using the High Sensitivity DNA Kit (Agilent). Following the quantitation, cleaned amplicons had been mixed and combined in equimolar ratios. Pair-end sequencing using the Illumina MiSeq system (Illumina, San Diego, USA) had been carried out at CIBIO (Center of Integrative Biology) – University of Trento (Trento, Italy).

### Illumina data analysis and sequences identification by QIIME2

Raw paired-end FASTQ files were demultiplexed using idemp (https://github.com/yhwu/idemp/blob/master/idemp.cpp) and imported into Quantitative Insights Into Microbial Ecology (Qiime2, version 2018.2). Sequences were quality filtered, trimmed, de-noised, and merged using DADA2^40^. Chimeric sequences had been identified and removed via the consensus method in DADA2. Representative bacterial sequences had been aligned with MAFFT and used for phylogenetic reconstruction in FastTree using plugins alignment and phylogeny^41, 42^. Alpha and beta diversity metrics had been calculated using the core-diversity plugin within QIIME2 and visualised by emperor^43^. For bacteria, beta diversity metric had been calculated using the beta-diversity plugin within QIIME2 and the—metric command was set on weighted_normalized_unifrac. For bacteria, taxonomic and compositional analyses were carried on by using plugins feature-classifier (https://github.com/qiime2/q2-feature-classifier). A pre-trained Naive Bayes classifier based on the Greengenes 13_8 99% Operational Taxonomic Units (OTUs) database which had been previously trimmed to the V4 region of 16S rDNA, bound by the 341F/805R primer pair, was applied to paired-end sequence reads to generate taxonomy tables. For fungi, sequences were classified to the species-level using a 97 or 99% threshold using UNITE dynamic classifier version 8.0 released for QIIME2 (UNITE QIIME release for Fungi. Version 18.11.2018. UNITE Community. https://doi.org/10.15156/BIO/786334). The data generated by MiSeq Illumina sequencing were deposited in the NCBI Sequence Read Archive (SRA) and are available under Ac. Number PRJNA588404 (https://www.ncbi.nlm.nih.gov/bioproject/PRJNA588404/https://www.ncbi.nlm.nih.gov/bioproject/PRJNA588404/).

### Volatiles determination

The chemical volatiles released during the cycles were measured following the method by Narduzzi et al.^7^. Briefly, two Tenax cartridges (147, Radiello, Supelco), pre-conditioned under nitrogen flow for 2 h at 300 °C, were inserted in their dedicated air filters and placed on the top of the wooden bars of the grass baths for 24 h, to passively collect the volatiles released by the fermentation process. After 24 h, each cartridge was sealed in its glass tube and stored in the dark at 4 °C until analysis. In July, the d6 sample had been lost.

The cartridges were desorbed using a Thermal Desorber (Unity 2, Markes) and desorbed gases were injected in a comprehensive GCxGC–TOF–MS (LECO Corporation, St. Joseph, MI, USA). The chromatograms were acquired and de-convoluted using the ChromaTOF software (Version 4.22; LECO) pre-processed using the package GCxGC Leco.analyser^7^ and normalized using the VSN algorithm^44^ through the web service NOREVA^45^.

### Statistical analysis

A normality test (Shapiro–Wilk W) was performed, as well as a nonparametric tests (Kruskal–Wallis) analyzing the day of collection as independent variables and the microbial plate counts as dependent variables. All the tests on plate counts were performed using the STATISTICA data analysis software system, version 9.1 (StatSoft, Inc. 2010 ww.statsoft.com).

Differences in diversity indices (OTUs number and Shannon diversity index) of different samples were tested by Kruskal–Wallis test by a plug-in implemented in QIIME2. The overall structural changes of bacterial and fungal communities were visualised by principal coordinates analysis (PCoA) based on Weighted Unifrac for bacterial and Jaccard Distance Matrix for fungi community. In order to aid the ordination of the samples across time, the day of sampling has been considered as a custom-axis in both PCoAs by means of a plug-in implemented in QIIME2. The statistical significance of communities among all samples was assessed via the non-parametric PERMANOVA (permutational multivariate analysis of variance) by means of plug-in implemented in QIIME2.

The heat map of the volatiles compounds dataset was built using Euclidean score distance and Ward clustering algorithm in R environment.

## Supplementary information

Supplementary Information.

## References

[1] Petraglia A, Bellisai B, Manica P, Fioravanti A (2009). Phytobalneotherapy (“Hay Baths”): between tradition and modern medicine. Presse Therm. Clim..

[2] Tenti S, Manica P, Galeazzi M, Fioravanti A (2013). Phytothermotherapy in fibromyalgia and osteoarthritis: Between tradition and modern medicine. Eur. J. Integr. Med..

[3] Miori R, Paolazzi G, Albertazzi R (2008). Phytothermotherapy with fermenting alpine grass in knee osteoarthritis: mid-long term results. Reumatismo.

[4] Talamucci P, Piemontese S, Coser P (1996). La gestione dell'erba per i “Bagni di fieno”. Risultati di un triennio di studi. Rep. CEALP.

[5] Fioravanti A, Bellisai B, Iacoponi F, Manica P, Galeazzi M (2011). Phytothermotherapy in osteoarthritis: a randomized controller clinical trial. J. Altern. Complement. Med..

[6] Wrighton KC (2008). A novel ecological role of the Firmicutes identified in thermophilic microbial fuel cells. ISME J..

[7] Narduzzi L (2018). Applying novel approaches for GC × GC-TOF-MS data cleaning and trends clustering in VOCs time-series analysis: following the volatiles fate in grass baths through passive diffusion sampling. J. Chromatogr. B.

[8] Ryckeboer J (2003). A survey of bacteria and fungi occurring during composting and self-heating processes. Ann. Microbiol..

[9] Insam H, de Bertoldi M, Díaz LF (2007). Microbiology of the composting process. Waste Management Series No 8. Compost Science and Technology.

[10] Woolford, M. K. Bacterial developments their implications for silage production and aerobic stability In *Biotechnology in the Feed Industry, Proceedings of the 14”’ Annual Symposium* (Nottingham University Press, 1998).

[11] Gerardi, M. H. The microbiology of anaerobic digesters. In *Fermentation* 43–50 (Wiley, New Jersey, 2003).

[12] Ferreira JA, Mahboubi A, Lennartsson PR, Taherzadeh MJ (2016). Waste biorefineries using filamentous ascomycetes fungi: present status and future prospects. Bioresour. Technol..

[13] Zhang J (2011). Effects of physico-chemical parameters on the bacterial and fungal communities during agricultural waste composting. Bioresour. Technol..

[14] Ghoul M, Mitri S (2016). The ecology and evolution of microbial competition. Trends Microbiol..

[15] Partanen P, Hultman J, Paulin L, Auvinen P, Romantschuk M (2010). Bacterial diversity at different stages of the composting process. BMC Microbiol..

[16] López-González JA (2015). Dynamics of bacterial microbiota during lignocellulosic waste composting: studies upon its structure, functionality and biodiversity. Bioresour. Technol..

[17] Karadag D (2013). Profiling of bacterial community in a full-scale aerobic composting plant. Int. Biodeter. Biodegr..

[18] Jurado M (2014). Exploiting composting biodiversity: study of the persistent and biotechnologically relevant microorganisms from lignocellulose-based composting. Bioresour. Technol..

[19] Taylor CR (2012). Isolation of bacterial strains able to metabolize lignin from screening of environmental samples. J. Appl. Microbiol..

[20] Braun U, Nakashima C, Crous PW (2013). Cercosporoid fungi (Mycosphaerellaceae) 1 species on other fungi Pteridophyta and Gymnospermae. IMA Fungus.

[21] Buzzini P, Turchetti B, Yurkov A (2018). Extremophilic yeasts: the toughest yeasts around?. Yeast.

[22] Sannino C, Tasselli G, Filippucci S, Turchetti B, Buzzini P, Buzzini P (2017). Yeasts in nonpolar cold habitats. Yeasts in Natural Ecosystems: Diversity.

[23] https://www.climatrentino.it/binary/pat_climaticamente/osservatorio_trentino_clima/Libro_CLIMA_.1355921619.pdf.

[24] Miao Y (2015). Genome-wide transcriptomic analysis of a superior biomass-degrading strain of *A-fumigatus *revealed active lignocellulose-degrading genes. BMC Genom..

[25] Singh S, Madlala AM, Prior BA (2003). *Thermomyces lanuginosus:* properties of strains and their hemicellulases. FEMS Microbiol. Rev..

[26] McHunu NP (2013). Xylanase Superproducer: genome sequence of a compost-loving thermophilic fungus thermomyces lanuginosus strain SSBP. Genome Announc..

[27] Tariq S (2019). A comprehensive review of the antibacterial, antifungal and antiviral potential of essential oils and their chemical constituents against drug resistant microbial pathogens. Microb Pathog..

[28] Kowalski R, Sugier D, Sugier P, Kołodziej B (2015). Evaluation of the chemical composition of essential oils with respect to the maturity of flower heads of *Arnica montana* L. and *Arnica chamissonis* Less. cultivated for industry. Ind. Crops Prod..

[29] Loughlin R, Gilmore BF, McCarron PA, Tunney MM (2008). Comparison of the cidal activity of tea tree oil and terpinen-4-ol against clinical bacterial skin isolates and human fibroblast cells. Lett. Appl. Microbiol..

[30] Stojanovic G, Palic R, Tarr CH, Reddy CM, Marinkovic O (2003). n-alkanes and fatty acids of *Hypericum perforatum, Hypericum maculatum* and *Hypericum olympicum*. Biochem. Syst. Ecol..

[31] Bicas JL, Fontanille P, Pastore GM, Larroche C (2010). A bioprocess for the production of high concentrations of R-(+)-a-terpineol from R-(+)-limonene. Process Biochem..

[32] Bicas JL, Neri-Numa IA, Ruiz AL, De Carvalho JE, Pastore GM (2011). Evaluation of the antioxidant and antiproliferative potential of bioflavors. Food Chem Toxicol..

[33] Bezerra-Rodrigues L (2016). Anti-inflammatory and antiedematogenic activity of the *Ocimum basilicum* essential oil and its main compound estragole: in vivo mouse models. Chem. Biol. Interact..

[34] Siddiquee S, Al Azad S, Abu Bakar F, Naher L, Vijay Kumar S (2015). Separation and identification of hydrocarbons and other volatile compounds from cultures of *Aspergillus niger* by GC–MS using two different capillary columns and solvents. J. Saudi Chem. Soc..

[35] Fincheira P, Parra L, Mutis A, Parada M, Quiroz A (2017). Volatiles emitted by Bacillus sp. BCT9 act as growth modulating agents on *Lactuca sativa* seedlings. Microbiol. Res..

[36] Kanj A, Abdallah N, Soubani AO (2018). The spectrum of pulmonary aspergillosis. Resp. Med..

[37] Baker GC, Smith JJ, Cowan DA (2003). Review and re-analysis of domain-specific 16S primers. J. Microbiol. Methods.

[38] Claesson MJ (2010). Comparison of two next-generation sequencing technologies for resolving highly complex microbiota composition using tandem variable 16S rRNA gene regions. Nucleic Acids Res..

[39] Gardes M, Bruns TD (1993). ITS primers with enhanced specificity for higher fungi and basidiomycetes: application to identification of mycorrhizae and rusts. Mol. Ecol..

[40] Callahan BJ (2016). DADA2: high-resolution sample inference from Illumina amplicon data. Nat. Methods.

[41] Katoh K, Standley DM (2013). MAFFT multiple sequence alignment software version 7: improvements in performance and usability. Mol. Biol. Evol..

[42] Price MN, Dehal PS, Arkin AP (2009). FastTree: computing large minimum evolution trees with profiles instead of a distance matrix. Mol. Biol. Evol..

[43] Vazquez-Baeza Y, Pirrung M, Gonzalez A, Knight R (2013). EMPeror: a tool for visualizing high-throughput microbial community data. GigaScience.

[44] Huber W, von Heydebreck A, Sueltmann H, Poustka A, Vingron M (2002). Variance stabilization applied to microarray data calibration and to the quantification of differential expression. Bioinformatics.

[45] Li B (2017). NOREVA: normalization and evaluation of MS-based metabolomics data. Nucleic Acids Res..

