# Supplementary material for: Microbial community dynamics in phyto-thermotherapy baths viewed through next generation sequencing and metabolomics approach
Source: Sci Rep. 2020 Oct 21;10:17931. doi: 10.1038/s41598-020-74586-9 (PMC7578836; doi:10.1038/s41598-020-74586-9)
Supplement: Supplementary file 1 — Supplementary Information. [file 41598_2020_74586_MOESM1_ESM.docx]

**Microbial community dynamics in phyto-thermotherapy baths viewed through next generation sequencing and metabolomics approach**

Elena Franciosi^1^*, Luca Narduzzi^1^, Antonella Paradiso^1^, Silvia Carlin^1^, Kieran Tuohy^1^, Alberto Beretta^2^ and Fulvio Mattivi^1,3^

^1^Research and Innovation Centre, AgriFood Quality and Nutrition Department, Fondazione Edmund Mach (FEM). Via E. Mach 1, 38010 San Michele all'Adige, Italy

^2^Ospedale San Raffaele - Milano, via Olgettina 60, 20132 Milano, Italy

^3^University of Trento, Department of Cellular, Computational and Integrative Biology – CIBIO, Via Sommarive, 9, 38123 Trento, Italy

*Corresponding author. Tel.: +39 (0)461 615117; fax: +39 (0)461 615200. *E-mail address:* elena.franciosi@fmach.it.

**Table S1:** Total number of reads after merging and quality trimming of raw data in each herb sample, in different days, months, positions (cm) and replicates (A, B and C) of the PTB process.

| **Month** | **Replicate** | **Position** | **day** | **Bacteria** | **Fungi** |
| --- | --- | --- | --- | --- | --- |
| July | A | 5 | 0 | 45,124 | 18,490 |
| July | A | 40 | 0 | 60,285 | 14,147 |
| July | B | 5 | 0 | 56,587 | 16,466 |
| July | B | 40 | 0 | 27,307 | 11,421 |
| July | C | 5 | 0 | 35,822 | 13,279 |
| July | C | 40 | 0 | 17,097 | 10,151 |
| July | A | 5 | 2 | 37,085 | 9,924 |
| July | A | 40 | 2 | 27,474 | 10,299 |
| July | B | 5 | 2 | 36,163 | 10,309 |
| July | B | 40 | 2 | 37,269 | 8,914 |
| July | C | 5 | 2 | 34,421 | 11,082 |
| July | C | 40 | 2 | 44,347 | 15,243 |
| July | A | 5 | 3 | 47,236 | 14,838 |
| July | A | 40 | 3 | 48,995 | 13,533 |
| July | B | 5 | 3 | 43,837 | 11,187 |
| July | B | 40 | 3 | 42,560 | 11,970 |
| July | C | 5 | 3 | 39,716 | 15,597 |
| July | C | 40 | 3 | 39,749 | 9,878 |
| July | A | 5 | 5 | 48,538 | 13,826 |
| July | A | 40 | 5 | 24,320 | 8,684 |
| July | B | 5 | 5 | 34,497 | 10,189 |
| July | B | 40 | 5 | 16,021 | 7,165 |
| July | C | 5 | 5 | 28,699 | 7,490 |
| July | C | 40 | 5 | 3,327 | 8,968 |
| July | A | 5 | 7 | 36,624 | 9,023 |
| July | A | 40 | 7 | 37,497 | 8,264 |
| July | B | 5 | 7 | 38,440 | 7,815 |
| July | B | 40 | 7 | 32,168 | 9,385 |
| July | C | 5 | 7 | 41,631 | 10,757 |
| July | C | 40 | 7 | 41,171 | 8,516 |
| August | A | 5 | 0 | 29,106 | 9,709 |
| August | A | 40 | 0 | 28,009 | 14,932 |
| August | B | 5 | 0 | 23,101 | 15,044 |
| August | B | 40 | 0 | 26,537 | 13,545 |
| August | C | 5 | 0 | 32,074 | 13,993 |
| August | C | 40 | 0 | 40,463 | 12,947 |
| August | A | 5 | 2 | 46,846 | 13,410 |
| August | A | 40 | 2 | 42,310 | 11,657 |
| August | B | 5 | 2 | 45,702 | 12,679 |
| August | B | 40 | 2 | 25,062 | 12,287 |
| August | C | 5 | 2 | 41,983 | 12,429 |
| August | C | 40 | 2 | 37,185 | 9,671 |
| August | A | 5 | 3 | 41,035 | 11,875 |
| August | A | 40 | 3 | 45,722 | 10,884 |
| August | B | 5 | 3 | 43,153 | 11,562 |
| August | B | 40 | 3 | 31,874 | 9,397 |
| August | C | 5 | 3 | 48,183 | 8,381 |
| August | C | 40 | 3 | 34,936 | 9,388 |
| August | A | 5 | 5 | 41,337 | 8,728 |
| August | A | 40 | 5 | 31,845 | 8,419 |
| August | B | 5 | 5 | 36,806 | 11,111 |
| August | B | 40 | 5 | 37,118 | 9,159 |
| August | C | 5 | 5 | 34,103 | 9,467 |
| August | C | 40 | 5 | 37,485 | 9,657 |
| August | A | 5 | 7 | 29,602 | 8,172 |
| August | A | 40 | 7 | 25,610 | 7,543 |
| August | B | 5 | 7 | 30,492 | 9,388 |
| August | B | 40 | 7 | 32,868 | 7,917 |
| August | C | 5 | 7 | 27,219 | 8,847 |
| August | C | 40 | 7 | 47,669 | 10,616 |
| October | A | 5 | 0 | 34,548 | 23,276 |
| October | A | 40 | 0 | 40,075 | 29,948 |
| October | B | 5 | 0 | 31,023 | 21,806 |
| October | B | 40 | 0 | 6,525 | 22,106 |
| October | C | 5 | 0 | 9,636 | 21,524 |
| October | C | 40 | 0 | 39,451 | 19,634 |
| October | A | 5 | 2 | 29,531 | 22,483 |
| October | A | 40 | 2 | 26,528 | 16,127 |
| October | B | 5 | 2 | 26,388 | 25,991 |
| October | B | 40 | 2 | 26,644 | 24,343 |
| October | C | 5 | 2 | 30,873 | 11,140 |
| October | C | 40 | 2 | 34,328 | 20,341 |
| October | A | 5 | 3 | 28,432 | 18,465 |
| October | A | 40 | 3 | 22,471 | 16,441 |
| October | B | 5 | 3 | 28,648 | 19,230 |
| October | B | 40 | 3 | 23,352 | 19,635 |
| October | C | 5 | 3 | 37,575 | 21,731 |
| October | C | 40 | 3 | 33,998 | 24,165 |
| October | A | 5 | 5 | 23,226 | 12,495 |
| October | A | 40 | 5 | 22,854 | 22,644 |
| October | B | 5 | 5 | 11,779 | 11,174 |
| October | B | 40 | 5 | 18,135 | 10,063 |
| October | C | 5 | 5 | 29,270 | 17,610 |
| October | C | 40 | 5 | 34,390 | 17,356 |
| October | A | 5 | 7 | 28,592 | 23,622 |
| October | A | 40 | 7 | 29,252 | 20,635 |
| October | B | 5 | 7 | 18,050 | 14,951 |
| October | B | 40 | 7 | 23,256 | 13,405 |
| October | C | 5 | 7 | 21,799 | 14,623 |
| October | C | 40 | 7 | 30,440 | 10,504 |

**Table S2:** List of the 295 VOCs present in all the samples

| **Number** | **Compound** |
| --- | --- |
| 1 | 1,1'-biphenyl, 4-methyl- |
| 2 | 1,2,3-trimethoxybenzene |
| 3 | 1,2-benzenedicarboxylic acid, dibutyl ester |
| 4 | 1,2-benzenedicarboxylic acid, diethyl ester |
| 5 | 1,2-propanedione, 1-phenyl- |
| 6 | 1,3,5,7-cyclooctatetraene |
| 7 | 1,3,5-trioxane, 2,4,6-trimethyl- |
| 8 | 1,3,6-octatriene, 3,7-dimethyl-, (e)- |
| 9 | 1,3-cyclohexadiene, 1-methyl-4-(1-methylethyl)- |
| 10 | 1,3-dioxolan-2-one, 4-methyl- |
| 11 | 1,3-pentanediol, 2,2,4-trimethyl- |
| 12 | 1,3-propanediol, 2,2-dimethyl- |
| 13 | 1,3-di-iso-propylnaphthalene |
| 14 | 1,3a-ethano-3ah-indene, 1,2,3,6,7,7a-hexahydro-2,2,4,7a-tetramethyl-, 1r-(1alpha,3aalpha,7aalpha)- |
| 15 | 1,4,8-cycloundecatriene, 2,6,6,9-tetramethyl-, (e,e,e)- |
| 16 | 1,4-benzenediol, 2,6-bis(1,1-dimethylethyl)- |
| 17 | 1,4-cyclohexadiene, 1-methyl-4-(1-methylethyl)- |
| 18 | 1,4-dioxan-2-ol |
| 19 | 1,4-methanoazulene, decahydro-4,8,8-trimethyl-9-methylene-, 1s-(1alpha,3abeta,4alpha,8abeta)- |
| 20 | 1,5-octadien-3-ol, (z)- |
| 21 | 1,6-cyclodecadiene, 1-methyl-5-methylene-8-(1-methylethyl)-, s-(e,e)- |
| 22 | 1,6-octadiene, 7-methyl-3-methylene- |
| 23 | 1-butanone, 1-phenyl- |
| 24 | 1-cyclohexene-1-carboxaldehyde, 4-(1-methylethyl)- |
| 25 | 1-cyclohexene-1-carboxylic acid, 2,6,6-trimethyl-, methyl ester |
| 26 | 1-decanol |
| 27 | 1-hexanol |
| 28 | 1-hexanol, 2-ethyl- |
| 29 | 1-isopropyl-2,5-dimethoxy-4-methylbenzene |
| 30 | 1-isopropyl-4-methyl-3-cyclohexen-1-ol |
| 31 | 1-iodo-2-methylundecane |
| 32 | 1-methoxy-2-vinylbenzene |
| 33 | 1-nonanol |
| 34 | 1-octen-3-ol |
| 35 | 1-octen-3-one |
| 36 | 1-pentanol, 3-ethyl-4-methyl-, (s)- |
| 37 | 1-propanamine |
| 38 | 1-phenoxypropan-2-ol |
| 39 | 1-tetradecene |
| 40 | 1-undecanol |
| 41 | 11-methyldodecanol |
| 42 | 1h-indene, 2,3-dihydro- |
| 43 | 1h-indene, 2,3-dihydro-1,2-dimethyl- |
| 44 | 1h-pyrrole-2-carboxaldehyde |
| 45 | 2 octenal |
| 46 | 2(10)-pinen-3-one, (1s,5s)-(-)- |
| 47 | 2(3h)-furanone, 5-butyldihydro- |
| 48 | 2(3h)-furanone, 5-ethyldihydro- |
| 49 | 2(3h)-furanone, 5-hexyldihydro- |
| 50 | 2(3h)-furanone, dihydro- |
| 51 | 2(3h)-furanone, 5-ethenyldihydro-5-methyl- |
| 52 | 2(5h)-furanone, 5-ethyl-, (r)- |
| 53 | 2,2-dimethylpropanoic acid, tridec-2-ynyl ester |
| 54 | 2,3-butanediol, s-(r*,r*)- |
| 55 | 2,4-cycloheptadien-1-one, 2,6,6-trimethyl- |
| 56 | 2,4-hexadienal |
| 57 | 2,4-nonadienal, (e,e)- |
| 58 | 2,5-dimethylpyrazine |
| 59 | 2,6,6-trimethylcyclohex-2-ene-1,4-dione |
| 60 | 2,6-diisopropylnaphthalene |
| 61 | 2,6-dimethyl-1,3,5,7-octatetraene, e,e- |
| 62 | 2-(1-pyrrolyl)ethanol |
| 63 | 2-(2-butoxyethoxy)ethyl acetate |
| 64 | 2-(2-methylpropyl)-3-(1-methylethyl)pyrazine |
| 65 | 2-allyl-1,4-dimethoxy-3-methyl-benzene |
| 66 | 2-butanone, 3-hydroxy- |
| 67 | 2-cyclohexen-1-one, 2-methyl-5-(1-methylethenyl)- |
| 68 | 2-cyclohexen-1-one, 3,5,5-trimethyl- |
| 69 | 2-cyclohexen-1-one, 3-methyl-6-(1-methylethyl)- |
| 70 | 2-ethyl-1-dodecanol |
| 71 | 2-furanmethanol, 5-ethenyltetrahydro-alpha,alpha,5-trimethyl-, cis- |
| 72 | 2-heptanol |
| 73 | 2-heptanone |
| 74 | 2-hexenal |
| 75 | 2-hydroxy-iso-butyrophenone |
| 76 | 2-methyl-1-undecanol |
| 77 | 2-nonanol |
| 78 | 2-nonanone |
| 79 | 2-norpinene, 2,6-dimethyl-6-(4-methyl-3-pentenyl)-, trans-(-)- |
| 80 | 2-octanone |
| 81 | 2-pentanone, 5-hydroxy- |
| 82 | 2-propanone |
| 83 | 2-propenoic acid, decyl ester |
| 84 | 2-propanol, 1-(2-methoxy-1-methylethoxy)- |
| 85 | 2-propanol, 1-hydrazino- |
| 86 | 2-pyrrolidinone, 1-methyl- |
| 87 | 2-undecanone |
| 88 | 2-undecanethiol, 2-methyl- |
| 89 | 2-undecenal |
| 90 | 2-n-butylacrolein |
| 91 | 2h-1-benzopyran-2-one, 3,4-dihydro- |
| 92 | 2h-azepin-2-one, hexahydro- |
| 93 | 2h-pyran-3(4h)-one, 6-ethenyldihydro-2,2,6-trimethyl- |
| 94 | 3,5-heptadien-2-one, 6-methyl-, (e)- |
| 95 | 3,5-octadien-2-one |
| 96 | 3,6,9,12-tetraoxatetradecane-1,14-diol |
| 97 | 3,6-heptanedione |
| 98 | 3,6-octadien-1-ol, 3,7-dimethyl-, (z)- |
| 99 | 3,7-dimethyl-1,6-octadien-3-ol |
| 100 | 3-buten-2-one, 4-(2-hydroxy-2,6,6-trimethylcyclohexyl)- |
| 101 | 3-decanone |
| 102 | 3-ethylcyclopentanone |
| 103 | 3-furaldehyde |
| 104 | 3-furylmethanol |
| 105 | 3-heptanone |
| 106 | 3-hexen-1-ol |
| 107 | 3-isobutyl-1-methyl-cyclopentanone |
| 108 | 3-methyl-2(5h)-furanone |
| 109 | 3-nonanone |
| 110 | 3-octanol |
| 111 | 3-octanone |
| 112 | 3-pinanone |
| 113 | 3-tridecanone |
| 114 | 3-tridecene, (e)- |
| 115 | 3-undecanone |
| 116 | 4-dodecene, (e)- |
| 117 | 4-hydroxy-3-methylacetophenone |
| 118 | 4-isopropylcyclohex-2-en-1-one |
| 119 | 4-isopropylcyclohexanone |
| 120 | 4-nonylphenol |
| 121 | 4-undecene, 5-methyl-, (e)- |
| 122 | 4-tert-butylcyclohexyl acetate |
| 123 | 5,9-dodecadien-2-one, 6,10-dimethyl-, (e,e))- |
| 124 | 5,9-undecadien-2-one, 6,10-dimethyl-, (e)- |
| 125 | 5-ethyl-2-heptanone |
| 126 | 5-hepten-2-one, 6-methyl- |
| 127 | 5-hepten-2-ol, 6-methyl- |
| 128 | 5-hexenal, 4-methylene- |
| 129 | 5-undecanone |
| 130 | 6-nonenal, (z)- |
| 131 | 6-nitroundec-5-ene |
| 132 | 9-octadecen-1-ol, (z)- |
| 133 | acetamide |
| 134 | acetic acid |
| 135 | azulene |
| 136 | azulene, 1,2,3,3a,4,5,6,7-octahydro-1,4-dimethyl-7-(1-methylethenyl)-, 1r-(1alpha,3abeta,4alpha,7beta)- |
| 137 | acenaphthene |
| 138 | acetaldehyde, tetramer |
| 139 | acetophenone |
| 140 | anisole |
| 141 | benzaldehyde, 2-hydroxy- |
| 142 | benzaldehyde, 4-(1-methylethyl)- |
| 143 | benzaldehyde, 4-methoxy- |
| 144 | benzene, (1,1-dimethylpropyl)- |
| 145 | benzene, (1-pentylheptyl)- |
| 146 | benzene, 1,2,3,4-tetramethyl- |
| 147 | benzene, 1,2,3-trimethyl- |
| 148 | benzene, 1,2,4,5-tetramethyl- |
| 149 | benzene, 1,2-dimethoxy- |
| 150 | benzene, 1,2-dimethoxy-4-(2-propenyl)- |
| 151 | benzene, 1,2-dimethyl- |
| 152 | benzene, 1,3-diethyl- |
| 153 | benzene, 1,3-dimethyl-5-(1-methylethyl)- |
| 154 | benzene, 1-ethenyl-4-methoxy- |
| 155 | benzene, 1-ethyl-2-methyl- |
| 156 | benzene, 1-ethyl-4-methoxy- |
| 157 | benzene, 1-ethyl-4-methyl- |
| 158 | benzene, 1-methoxy-4-methyl- |
| 159 | benzene, 1-methyl-3-propyl- |
| 160 | benzene, 1-methyl-4-(1-methylethyl)- |
| 161 | benzene, 2-ethyl-1,3-dimethyl- |
| 162 | benzene, butyl- |
| 163 | benzene, ethyl- |
| 164 | benzene, propyl- |
| 165 | benzeneacetaldehyde |
| 166 | benzenemethanol |
| 167 | benzenemethanol, alpha,alpha,4-trimethyl- |
| 168 | benzenemethanol, alpha,alpha-dimethyl- |
| 169 | benzenemethanol, alpha-methyl- |
| 170 | benzoic acid |
| 171 | benzoic acid, 2-hydroxy-, methyl ester |
| 172 | benzonitrile |
| 173 | bicyclo2.2.1heptan-2-ol, 1,7,7-trimethyl-, (1s-endo)- |
| 174 | bicyclo3.1.0hexane, 4-methylene-1-(1-methylethyl)- |
| 175 | bicyclo3.1.1hept-2-ene, 2,6,6-trimethyl- |
| 176 | bicyclo7.2.0undec-4-ene, 4,11,11-trimethyl-8-methylene-, 1r-(1r*,4e,9s*)- |
| 177 | butanoic acid |
| 178 | butanoic acid, 2-methyl-, methyl ester |
| 179 | butanoic acid, 3-methyl- |
| 180 | benzaldehyde, 4-hydroxy- |
| 181 | benzamide |
| 182 | benzene, (1-ethyloctyl)- |
| 183 | benzene, (1-methylethyl)- |
| 184 | benzene, (1-propyldecyl)- |
| 185 | benzene, 1,3-diethyl-5-methyl- |
| 186 | benzene, 1,4-dimethoxy- |
| 187 | benzene, 1,4-dimethyl-2-(2-methylpropyl)- |
| 188 | benzene, 1-methoxy-2-methyl- |
| 189 | benzene, 1-methyl-4-(1-methylpropyl)- |
| 190 | benzene, 1-methyl-4-(2-methylpropyl)- |
| 191 | benzene, 2,4-diethyl-1-methyl- |
| 192 | benzene, 2,4-dimethyl-1-(1-methylpropyl)- |
| 193 | benzene, 2-methoxy-4-methyl-1-(1-methylethyl)- |
| 194 | benzene, 3-cyclohexen-1-yl- |
| 195 | benzoic acid, methyl ester |
| 196 | bicyclo2.2.1heptan-2-one, 1,7,7-trimethyl-, (1s)- |
| 197 | bicyclo3.1.0hexan-2-one, 5-(1-methylethyl)- |
| 198 | bicyclo3.1.1hept-3-en-2-one, 4,6,6-trimethyl-, (1s)- |
| 199 | bicyclo3.1.1heptan-2-one, 6,6-dimethyl-, (1r)- |
| 200 | biphenyl |
| 201 | butanamide, 3-methyl- |
| 202 | butanoic acid, 2,3-dimethyl-, methyl ester |
| 203 | butyl benzoate |
| 204 | cyclohexanol, 1-methyl-4-(1-methylethenyl)-, acetate |
| 205 | cyclohexanol, 5-methyl-2-(1-methylethyl)-, (1alpha,2alpha,5beta)- |
| 206 | cyclohexanone |
| 207 | cyclohexene, 1-methyl-4-(1-methylethenyl)- |
| 208 | cyclohexene, 1-methyl-4-(1-methylethenyl)-, (s)- |
| 209 | cyclohexene, 3-methylene-6-(1-methylethyl)- |
| 210 | cyclopropanoic acid -2,2-dimethyl-3-(2,2-dimethylethenyl), methyl ester |
| 211 | cyclotetradecane |
| 212 | cyclobutanecarboxylic acid, 2-pentadecyl ester |
| 213 | cyclohept-4-enone |
| 214 | cyclohexane, decyl- |
| 215 | cyclohexene, 1-methyl-4-(1-methylethylidene)- |
| 216 | cyclopentanone, 2,4,4-trimethyl- |
| 217 | cyclopropene, 1-butyl-2-ethyl- |
| 218 | decanal |
| 219 | decanoic acid |
| 220 | decanoic acid, methyl ester |
| 221 | dibenzofuran |
| 222 | diethyl ethylphosphonate |
| 223 | docosane |
| 224 | dodecane |
| 225 | decane, 3-methyl- |
| 226 | dehydromevalonic lactone |
| 227 | ethanol, 2,2'-oxybis- |
| 228 | ethanol, 2-(2-ethoxyethoxy)- |
| 229 | ethanol, 2-butoxy- |
| 230 | ethanone, 1-(4-methyl-3-cyclohexen-1-yl)- |
| 231 | estragole |
| 232 | ethanol, 2-(2-butoxyethoxy)- |
| 233 | ethanol, 2-phenoxy- |
| 234 | ethanone, 1,2-diphenyl- |
| 235 | ethyl 2-cyano-3-methyl-2-(o-methylbenzyl)butanoate |
| 236 | formamide |
| 237 | formamide, n,n-dimethyl- |
| 238 | formic acid |
| 239 | furan, 2,5-dihydro-2,2,4-trimethyl- |
| 240 | furan, 2-pentyl- |
| 241 | furan, 3-(4-methyl-3-pentenyl)- |
| 242 | furfural |
| 243 | heneicosane |
| 244 | hexadecane |
| 245 | hexanal |
| 246 | hexanoic acid |
| 247 | hexanoic acid, 2-hexenyl ester, (e)- |
| 248 | heptadecane, 2,6-dimethyl- |
| 249 | hexanoic acid, 2-ethyl- |
| 250 | methane, sulfinylbis- |
| 251 | methane, sulfonylbis- |
| 252 | methanone, diphenyl- |
| 253 | methyl 3-methylbutanoate |
| 254 | n,n-dibutylformamide |
| 255 | naphthalene, 1,2,3,4-tetrahydro- |
| 256 | naphthalene, 1,2,3,4-tetrahydro-1,1,6-trimethyl- |
| 257 | naphthalene, 1-methyl- |
| 258 | naphthalene, 2-methoxy- |
| 259 | nonanal |
| 260 | nonane |
| 261 | naphthalene, 1,7-dimethyl- |
| 262 | naphthalene, 1-ethyl- |
| 263 | octanal |
| 264 | octanoic acid |
| 265 | octyl formate |
| 266 | pentanoic acid |
| 267 | pentanoic acid, 2-methyl- |
| 268 | phenol, 2-methoxy- |
| 269 | phenol, 2-methyl- |
| 270 | phenol, 2-methyl-5-(1-methylethyl)- |
| 271 | propanoic acid, 2,2-dimethyl- |
| 272 | propanoic acid, 2-methyl- |
| 273 | pentanol, 5-amino- |
| 274 | phenol, 2,4-bis(1,1-dimethylethyl)- |
| 275 | phenol, p-tert-butyl- |
| 276 | phenylethyl alcohol |
| 277 | propane, 1,2-dimethoxy- |
| 278 | propanoic acid, 2-methyl-, 2,2-dimethyl-1-(2-hydroxy-1-methylethyl)propyl ester |
| 279 | propanoic acid, 2-methyl-, 3-hydroxy-2,4,4-trimethylpentyl ester |
| 280 | sulfur dioxide |
| 281 | styrene |
| 282 | tetradecane |
| 283 | tridecane, 3-methyl- |
| 284 | triacetin |
| 285 | tridecane, 3-methylene- |
| 286 | undecane |
| 287 | undecanoic acid, methyl ester |
| 288 | cis-thujopsene |
| 289 | o-cymene |
| 290 | o-hydroxybiphenyl |
| 291 | p-nitrophenyl hexanoate |
| 292 | trans-2-(2-pentenyl)furan |
| 293 | alpha,beta-crotonolactone |
| 294 | alpha-terpineol |
| 295 | beta-copaene |
